# Level of Awareness and Attitude Toward Cerebral Palsy Among Parents in Al-Baha City, Saudi Arabia

**DOI:** 10.7759/cureus.31791

**Published:** 2022-11-22

**Authors:** Elfatih M Salih, Saleem A Alghamdi, Rayan A Alghamdi, Mohannad S Alghamdi, Turki A Alzahrani

**Affiliations:** 1 Pediatrics, University of Dongola, Dongola, SDN; 2 Pediatrics, Albaha University, Al-Baha, SAU; 3 Anesthesia, King Fahad Medical City, Riyadh, SAU; 4 Orthopedics, King Fahad Hospital, Jeddah, SAU; 5 Psychiatry, King Abdulaziz Hospital, Jeddah, SAU; 6 Anesthesia, King Faisal Specialist Hospital & Research Centre, Jeddah, SAU

**Keywords:** attitude, awareness, knowledge, parents, cerebral palsy

## Abstract

Background

Cerebral palsy (CP) is a chronic disorder of motion, posture, and tone which occurs due to brain insult during the period of brain growth. It is a disabling disorder in both motor and intellectual aspects. Fortunately, CP is a manageable disease that can be managed in part by increasing the knowledge and understanding of the parents.

Methodology

This cross-sectional, prospective, community-based study aimed to assess the level of parents’ knowledge and their attitude toward CP using an electronic questionnaire. The parents’ knowledge was classified as good or poor based on an adopted scoring system. The parents’ attitude was categorized as positive or negative.

Results

Our study results showed that good knowledge (those with a score more than 60% of the total score) was noted in 275 (61.1%) participants, whereas 175 of the participants had poor knowledge (38.9%), especially regarding awareness of the diagnosis of CP.

Conclusions

Most participants had an overall good knowledge of CP although they had insufficient knowledge of some aspects of the disease such as causes, disease course, clinical presentations, diagnosis, and prognosis. Although the results showed a positive attitude concerning playing with a child with CP, unfortunately, there was a negative attitude toward hiring a CP patient and a strongly negative attitude toward marrying a patient with CP.

## Introduction

Cerebral palsy (CP) is defined as a permanent disorder of movement and posture, leading to difficulty in motion, which occurs due to a non-progressive brain insult during brain development [1]. Globally, the prevalence of CP is estimated to be 2-2.5 children per 1,000 live births [2]. In Saudi Arabia, limited data exist concerning the prevalence of CP. One study by Al Salloum et al. showed that CP is the most common neurological disorder among Saudi children with a prevalence rate of 2.34/1,000 live births [3].

There are multiple etiologies for CP which include genetic causes that affect intrauterine fetal brain development, congenital malformation, and intrauterine infections such as rubella and cytomegalovirus. Other causes include birth asphyxia, low birth weight, twin pregnancy, neonatal stroke, and jaundice [4].

CP is classified according to the predominant motor characteristics as spastic, hypotonic, athetotic, dystonic, and ataxic. According to the topographical involvement of the limbs, CP is classified as monoplegia, diplegia, triplegia, hemiplegia, or quadriplegia. Another method classifies CP based on the site of the lesion as pyramidal and extrapyramidal [5]. Diagnosis of CP is primarily clinical by a history of risk factors together with physical examination findings. However, magnetic resonance imaging (MRI) and other imaging studies can be used as an adjuvant to confirm brain insult if there is no obvious etiology for the patient’s symptoms [6,7]. Together with motor problems, children with CP may have associated speech problems, learning difficulties, seizure disorders, and visual or hearing impairments, in addition to behavioral and emotional problems [8].

Evaluation of children with CP should be ideally performed by a multispecialty team [9]. Management of children with CP primarily focuses on medical care and rehabilitation to improve their quality of life and enhance the improvement of social and motor skills. This can be achieved by referring the child to a multidisciplinary child development center [10,11].

Most children with spastic diplegia have normal cognitive aspects and a favorable prognosis for independent ambulation, while those with spastic quadriplegia are most likely to have significant functional limitations, impaired cognition, seizure disorder, and visual impairment; therefore, these children usually have a bad prognosis for self-ambulation.

Most children with spastic hemiplegia have a normal cognitive function and can maintain autonomous ambulation, while those with dyskinetic CP have an increased incidence of comorbid problems such as impaired cognition, epilepsy, behavioral problems, sleep disorders, and visual or hearing problems [12].

## Materials and methods

Study design, period, and area

This prospective, cross-sectional, community-based study was conducted from March 2022 to July 2022 to evaluate the level of parents’ awareness toward CP in Al-Baha, the capital city of the Al-Baha area located in southwest Saudi Arabia.

Data collection

An electronic questionnaire was designed in Arabic language covering the specific objectives of the study. It was distributed electronically to the target population (parents with children aged less than 17 years). These parents were encouraged to fill out the questionnaire after getting their acceptance for participation through informed consent. The sample size was calculated using the website Sample Size Calculator at a confidence level of 95% and a 5% margin of error. The population of Al-Baha city is about 400,000. After obtaining the minimum target sample size (385), we included as many eligible participants as possible who agreed to participate during the study period (five months).

Analytical methods

The data were analyzed using SPSS Statistics version 25 (IBM Corp., Armonk, NY). After retranslation of the questionnaire back to the English language and coding the variables, data were considered to be statistically significant at a p-value of ≤0.05.

Knowledge Score System

A scoring system was adopted from a previous study [13] to evaluate the knowledge of participants who were included in the study. Aspects of knowledge evaluation include questions about cause, progression, association with spasticity, flaccidity, developmental delay, intellectual and speech problems, seizures, effect on school performance, schools suitable for learning for a child with CP, diagnosis, management of CP, and whether it is curable. The correct response was given one while the incorrect response scored zero. A cut-off point of 60% was used to classify the responses as good and poor knowledge. Scores more than 60% were considered good knowledge, whereas those equal to or less than 60% were considered poor knowledge.

Ethical considerations

Ethical approval for the study was obtained from the Ethical Committee of the Faculty of Medicine, Al-Baha University (approval number: REC/PEA/BU-FM/2022/4). Informed consent was included in the questionnaire to obtain the participant’s acceptance electronically before filling out the questionnaire. The privacy of the participants was ensured, and they were reassured that their data will be used only for research legally and ethically.

## Results

We received a total of 450 responses. All of the 450 participants gave consent and answered all items in the questionnaire. The sociodemographic characteristics of the interviewed parents are shown in Table 1. Overall, 62.7% were male (fathers) and 37.3% were female parents. The age distribution showed that the majority of the respondents (36.7%) belonged to the age group of 35-44 years, and the level of education revealed that 76% had university or above educational qualifications. The majority of the respondents (70.2%) were employed, and 29.8% were unemployed (Table 1).

**Table 1:** Demographic characteristics.

Factor	N = 450	%
Relationship to child		
Father	282	62.7
Mother	168	37.3
Age (years)		
15–24	66	14.7
25–34	108	24
35–44	165	36.7
45–55	76	16.9
Older than 55	35	7.8
Educational level		
Elementary school	26	5.8
High school	82	18.2
University	288	64
Higher education	54	12
Socioeconomic status		
Poor	22	4.9
Middle	388	86.2
High	40	8.9
Employment status		
Employed	316	70.2
Unemployed	134	29.8

Only about one-third of the participants (36%) had not heard about CP, and only 4% had a family member affected with CP.

**Figure 1:**
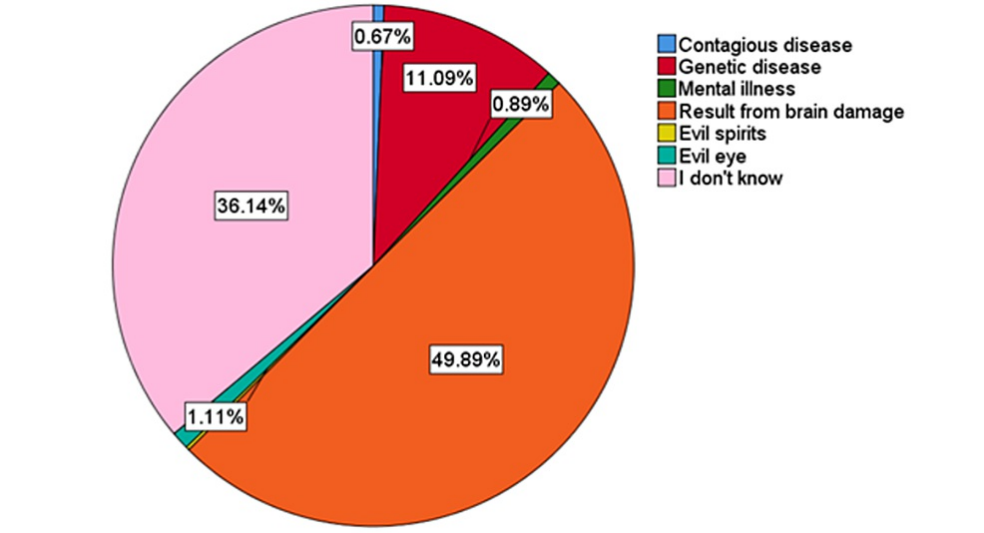
Participants’ view about the causes of cerebral palsy.

On reviewing the participants’ awareness concerning the cause of CP about, 1% thought it was a contagious disease, the same percentage referred to it as mental illness, about 11% said that it was a genetic disease, while 50% of the participants attributed it to brain injury or insult. Six participants thought that it was caused by an evil spirit or evil eye, and 36% did not know the cause of CP (Figure 1).

On assessing the knowledge of the participants concerning the course and progression of CP, 62% did not know if it was a static or progressive disease, while 22% said that it was a progressive disease, and only 16% thought that it was static.

On assessing the participants’ awareness regarding the characteristics and common presentations of CP, 55% of the respondents did not know whether CP is associated with muscle stiffness (spasticity), while 45% reported that spasticity often associates with CP. Two-thirds of the participants approved the association of CP with developmental delay, difficulty in understanding, and impaired cognition, while the remaining one-third had no idea about this association. We also found that 71% of the participants assumed that CP is associated with speech problems while 29% did not know about this association. In response to a question about whether CP is accompanied by seizure disorder, 63% of the parents said yes and 2% responded with no, while 35% did not know that CP is associated with convulsion. Overall, 10% thought that a child with CP can be taught in ordinary schools, while 90% said that they need special schools (Table 2).

**Table 2:** Participants’ awareness regarding the characteristics and clinical features of CP. CP: cerebral palsy

Number	Questions	Elementary	High school	University	Higher education	Total	P-value
1	Hearing about CP						0.510
	Yes	17 (65.4)	49 (59.8)	182 (63.2)	39 (72.2)	287 (63.8)	
	No	9 (34.6)	33 (40.2)	106 (36.8)	15 (27.8)	163 (36.2)	
2	CP is often associated with muscle stiffness						0.619
	Yes	8 (30.8)	36 (43.9)	134 (46.5)	26 (48.1)	204 (45.3)	
	No	2 (7.7)	3 (3.7)	16 (5.6)	1 (1.9)	22 (4.9)	
	Don’t know	16 (61.5)	43 (52.4)	138 (47.9)	27 (50)	224 (49.9)	
3	CP is often associated with developmental delay						0.113
	Yes	13 (50)	49 (59.8)	192 (66.7)	41 (75.9)	295 (65.6)	
	No	1 (3.8)	1 (1.2)	3 (1)	2 (3.7)	7 (1.5)	
	Don’t know	12 (46.2)	32 (39)	93 (32.3)	11 (20.4)	148 (32.9)	
4	CP may be accompanied by difficulty in understanding and perceiving						0.219
	Yes	13 (50)	51 (62.2)	200 (69.4)	37 (68.5)	301 (66.9)	
	No	2 (7.7)	4 (4.9)	11 (3.8)	5 (9.3)	22 (4.9)	
	Don’t know	11 (42.3)	27 (32.9)	77 (26.8)	12 (22.2)	127 (28.2)	
5	CP may be accompanied by difficulty speaking						0.685
	Yes	17 (65.4)	55 (67.1)	208 (72.2)	40 (74)	320 (71.1)	
	No	0 (0)	1 (1.2)	5 (1.8)	2 (3.7)	8 (1.8)	
	Don’t know	9 (34.6)	26 (31.7)	75 (26)	12 (22.3)	122 (27.1)	
6	Some types of CP may be accompanied by seizure						0.163
	Yes	10 (38.5)	49 (59.8)	186 (64.6)	38 (70.3)	283 (62.9)	
	No	1 (3.8)	1 (1.2)	4 (1.4)	1 (1.9)	7 (1.5)	
	Don’t know	15 (57.7)	32 (39)	98 (34)	15 (27.8)	160 (35.6)	
7	Teaching and learning of a child with CP						0.527
	In regular classes	4 (15.4)	9 (11)	27 (9.4)	3 (5.6)	43 (9.6)	
	Special needs classes	22 (84.6)	73 (89)	261(90.6)	51 (94.4)	407 (90.4)	
8	CP is a curable disease						0.001
	Yes	4 (15.4)	18 (22)	30 (10.4)	5 (9.3)	57 (12.7)	
	No	2 (7.7)	19 (23.2)	83 (28.8)	26 (48.1)	130 (28.9)	
	Don’t know	20 (76.9)	45 (54.8)	175 (60.8)	23 (42.6)	263 (58.4)	

Regarding the diagnosis of CP, 68% thought that brain radiological imaging is important for its diagnosis, while 2% stated that it is not required for diagnosis, and 30% answered with do not know. On checking participants’ knowledge about whether CP treatment should be carried out by a single doctor or team, 50% did not know the answer, while 44% thought that its management needs multiple doctors. On reviewing the respondents’ knowledge about the prognosis of CP, 12% thought that it is a curable disease, while 29% assumed that it is an uncurable disease, and 58% did not know whether it is a curable disease (Table 2).

**Figure 2:**
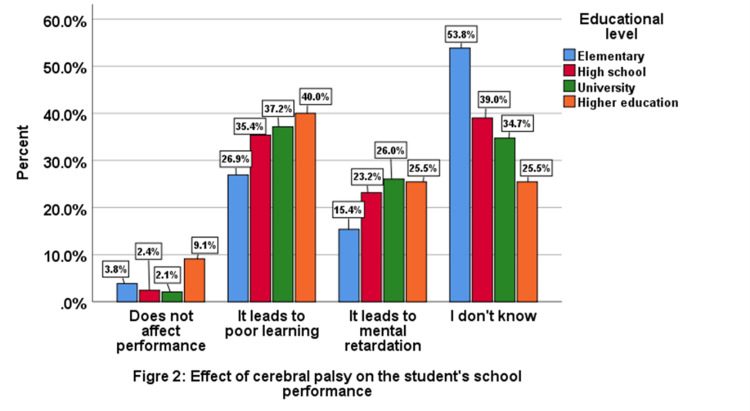
Effect of cerebral palsy on student’s school performance.

On checking the participants’ knowledge about the effect of CP on the student’s school performance 36.7% stated that it leads to poor school performance, 25% thought that it leads to mental retardation, 3% thought that it does not affect school performance, and 35.6% answered with do not know (Figure 2).

The knowledge score of the participants was compared to their level of education, and the results are shown in Table 3.

**Table 3:** Participants’ knowledge compared to their educational level.

Educational level	Poor knowledge	Total	%	Good knowledge	Total	%
Elementary	17	50/107	46.7	9	57/107	53.3
High school	34			48		
University	109	124/343	36.2	179	219/343	63.8
Higher education	15			40		

The attitude of participants toward CP patients was assessed based on their level of education and socioeconomic status, and the results are presented in Table 4.

**Table 4:** Participants’ attitudes in different educational levels and socioeconomic classes.

Attitude vs. educational level							
Number	Item	Elementary	High school	University	Higher education	Total	P-value
1	Employing a CP patient						0.02
	Yes	11 (42.3)	32 (39)	151 (52.4)	35 (64.8)	229 (50.9)	
	No	15 (57.7)	50 (61)	137 (47.6)	19 (35.2)	221 (49.1)	
2	Marrying a CP patient						0.797
	Yes	5 (19.3)	10 (12.2)	45 (15.6)	9 (16.7)	69 (15.3)	
	No	21 (80.8)	72 (87.8)	243 (84.4)	45 (83.3)	381 (84.7)	
3	Playing with a CP patient						0.274
	Yes	21 (80.8)	66 (80.5)	253 (87.8)	48 (88.9)	388 (86.2)	
	No	5 (19.3)	16 (19.5)	35 (12.2)	6 (11.1)	62 (13.8)	
Attitude vs. socioeconomic status							
Number	Item	Low	Middle	High	Total		P-value
1	Employing a CP patient						0.341
	Yes	8 (36.4)	199 (51.3)	22 (55)	229 (50.9)		
	No	14 (63.6)	189 (48.7)	18 (45)	221 (49.1)		
2	Marrying a CP patient						0.667
	Yes	2 (9.1)	60 (15.5)	7 (17.5)	69 (15.3)		
	No	20 (90.9)	328 (84.5)	33	381 (84.7)		
3	Playing with a CP patient						0.006
	Yes	14 (63.6)	338 (87.1)	36 (90)	388 (86.2)		
	No	8 (36.4)	50 (12.9)	4 (10)	62 (13.8)		

The analysis of the source of information related to CP showed that the most common source was reading (38.1%), followed by information from friends and family (25%), social media (13.8%), doctors and/or medical staff (12.3%), and education (10.8%).

## Discussion

Assessing the current level of knowledge and attitude of parents toward CP is crucial for the planning of educational strategies to improve and raise the health education of the community, which, in turn, will help in decreasing the incidence of the disease.

Our results showed that most of our participants had poor knowledge of specific aspects of CP, namely, its causes and risk factors. This is consistent with a study conducted in Indonesia [14] and another study conducted among pregnant women in Poland [15]. Results also showed poor knowledge about the course of the disease (progressive or static), clinical presentation, diagnosis, and prognosis. Public understanding of these aspects of the disease is of crucial importance.

The maximum possible correct score in knowledge-specified questions was 13. The obtained scores among the participants ranged between 4 and 13, with a mean score of 8.5. Good knowledge (more than 60% of the total score) was found in 275 (61.1%), whereas 175 (38.9%) participants had poor knowledge. This is more or less comparable to a study done in Nigeria [16] which found that 71% of the participants have good knowledge.

Studying the participants’ knowledge in comparison with their level of education showed that knowledge and awareness toward CP among participants with university or higher educational levels were better than in those with primary and secondary school education.

There was a significant association between the participants’ knowledge and their level of education. This was evident by the finding that 63.8% of the participants with university or higher education had good knowledge and 36.2% had poor knowledge. While among those with primary and secondary school education, 53.3% had good knowledge and 46.7% had knowledge. This finding is consistent with the findings of the study by Ashkanani and Al-Sane in Kuwait [17].

We also studied the attitude of the participants toward CP comparing it to their level of education and socioeconomic status. We found a high percentage of negative attitudes among participants with low educational levels (elementary and secondary school) and low socioeconomic status toward employing a patient with CP in their business while this percentage was reversed in those with high educational levels (university, higher education) and high socioeconomic status. This difference in the attitude of the participants in relation to their educational and socioeconomic level was found to be statistically significant (p = 0.02), which indicates that a good attitude is always paralleled with good knowledge and education. This finding contradicts the observation of Priyadharishini et al. [18] who found no correlation between knowledge, attitude, and skills prior to the implementation of the interventional package, as well as the study by Abdulwahab and Al-Gain [19] who found that educational degrees had no important effect on the participants’ attitudes toward disabled people; however, it was consistent with another study [20]. While there was a negative attitude toward marrying a family member to a patient with CP among most participants irrespective of their level of education or socioeconomic status. This may be attributed to the nature of CP being a lifelong disease in addition to the expected physical and intellectual dysfunctions which lead to fear about the future of such a marriage. Whereas there was a high percentage of positive attitudes (86.2%) among respondents regardless of their level of education and socioeconomic status when they were asked if they would let their children play with a child with CP, which was found to be statistically significant (p = 0.006). This was similar to what Sillanpää reported in a nationwide study [20]. Obviously, this is because playing will not lead to consequent harm.

## Conclusions

Most participant parents in this study in the Al-Baha area have an overall good knowledge of CP, although their knowledge was insufficient or poor in some aspects of the disease such as causes, disease course, clinical presentations, diagnosis, and prognosis. However, there was a positive attitude concerning letting their child play with a child with CP, a negative attitude toward hiring a patient with CP, and a strongly negative attitude toward marrying a patient with CP. These issues and aspects of poor knowledge need to be addressed and educational programs and workshops to be regulated to fill the gaps in knowledge and improve community awareness about this condition, which, in turn, can help in reducing its incidence and help parents deal properly with their children with CP. In addition, improving knowledge will improve the public attitude toward patients with CP.

## References

[1] Novak I, Morgan C, Adde L (2017). Early, accurate diagnosis and early intervention in cerebral palsy: advances in diagnosis and treatment. JAMA Pediatr.

[2] Mushta SM, Khandaker G, Power R, Badawi N (2019). Cerebral palsy in the Middle East: epidemiology, management, and quality of life. Handbook of Healthcare in the Arab World.

[3] Al Salloum AA, El Mouzan MI, Al Omar AA, Al Herbish AS, Qurashi MM (2011). The prevalence of neurological disorders in Saudi children: a community-based study. J Child Neurol.

[4] Alruwaished A, Ali B, Alhowaimil L, Alhowaimil LA, Alhowaimil AA, Alhowaimil NA, Alessa A (2020). Knowledge and attitude of caregivers of cerebral palsy children in Riyadh city. Int J Med Dev Ctries.

[5] Jones MW, Morgan E, Shelton JE, Thorogood C (2007). Cerebral palsy: introduction and diagnosis (part I). J Pediatr Health Care.

[6] Vitrikas K, Dalton H, Breish D (2020). Cerebral palsy: an overview. Am Fam Physician.

[7] Wood E (2006). The child with cerebral palsy: diagnosis and beyond. Semin Pediatr Neurol.

[8] Al-Dababneh KA, Al-Zboon EK (2018). Parents’ attitudes towards their children with cerebral palsy. Early Child Dev Care.

[9] Khalil M, Elweshahy H, Abdelghani H, Omar T, Ahmed S (2018). Quality of care provided to children with cerebral palsy, Alexandria, Egypt. East Mediterr Health J.

[10] Keys M, Lewis C (2019). An interdisciplinary approach for treating children with cerebral palsy. Online J Interprof Health Promotion.

[11] Chaplin S (2017). Assessment and management of cerebral palsy in the under-25s. Prescriber.

[12] Patel DR, Neelakantan M, Pandher K, Merrick J (2020). Cerebral palsy in children: a clinical overview. Transl Pediatr.

[13] Mohammed S, Omer IM (2005). Knowledge, Attitude and Practice of Mothers of Children With Cerebral Palsy. https://core.ac.uk/download/pdf/71668845.pdf.

[14] Moenardi MC, Sungkar E, Hawani D (2020). Cerebral palsy parents’ knowledge, attitude, and behavior at Dr. Hasan Sadikin General Hospital Bandung 2014. Althea Med J.

[15] Lewicka M, Wdowiak A, Sulima M, Bakalczuk G, Nieściór B, Iłżecka J (2012). Knowledge of factors predisposing to the occurrence of cerebral palsy among pregnant women. J Pre-Clin Clin Res.

[16] Adegbemigun OD, Hamzat TK, Olaleye OA (2019). Knowledge and beliefs of informal caregivers of children with cerebral palsy in Nigeria concerning cerebral palsy. Med J Zambia.

[17] Ashkanani F, Al-Sane M (2013). Knowledge, attitudes and practices of caregivers in relation to oral health of preschool children. Med Princ Pract.

[18] Priyadharishini J, Sadan V, Prashanth Prashanth, Sebastian T (2017). Community-based intervention package on knowledge, attitude and selected skills of home care among primary care givers of children with cerebral palsy. Indian J Cont Nsg Edn.

[19] Abdulwahab SS, Al-Gain SI (2003). Attitudes of Saudi Arabian health care professionals towards people with physical disabilities. Asia Pacific Disabil Rehabil J.

[20] Sillanpää ML (1990). Public awareness of and attitudes toward cerebral palsy in a nation-wide adult population. Acta Paediatr Scand.

